# Protocol for assessing visual stimulus discrimination in *Danionella cerebrum* and *Danio rerio* using habituation/dishabituation

**DOI:** 10.1016/j.xpro.2026.104378

**Published:** 2026-02-19

**Authors:** Mirko Zanon, Davide Potrich, Andrea Messina, Giorgio Vallortigara

**Affiliations:** 1 Center for Mind/Brain Sciences, University of Trento, Rovereto, Italy

**Keywords:** Model Organisms, Cognitive Neuroscience, Behavior

## Abstract

Here, we present a protocol to assess visual discrimination in fish, specifically *Danionella cerebrum* and *Danio rerio*. We describe steps for the familiarization of subjects to reduce contextual novelty and habituation through repeated presentation of a constant feature stimulus. We then detail procedures for a dishabituation session that introduces a stimulus change. In this protocol, the recovery of behavioral response provides a measure of change detection and feature discrimination.

For complete details on the use and execution of this protocol, please refer to Zanon et al.^1^

## Highlights

• Guidance on familiarizing fish with the testing context to stabilize baseline behavior
• Steps to induce habituation to a target feature while varying noninformative cues
• Instructions for introducing a controlled change in the target feature at dishabituation
• Guidance on quantifying response recovery as a measure of visual discrimination

## Abstract (editor highlights)

Publisher’s note: Undertaking any experimental protocol requires adherence to local institutional guidelines for laboratory safety and ethics.

## Graphical abstract

**Figure:**
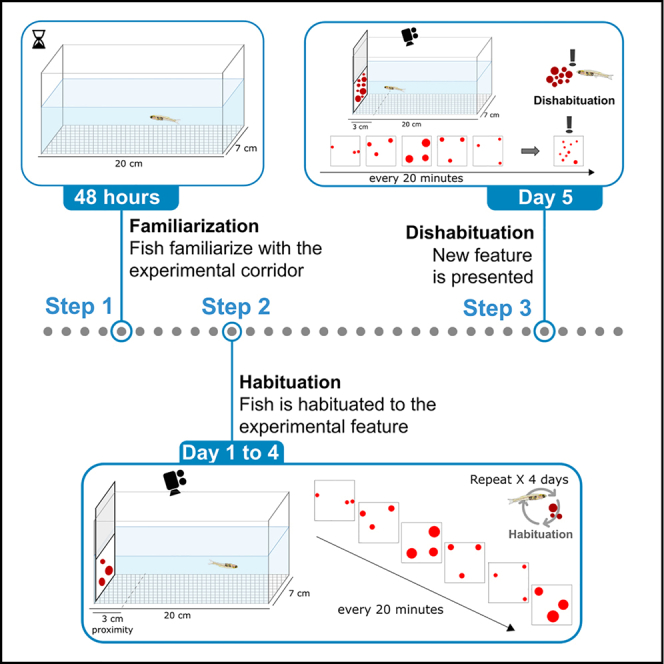

## Before you begin

The protocol below describes the procedure as applied in *Danionella* experiments on numerosity discrimination.^1^ A conceptually analogous habituation/dishabituation design has also been successfully implemented in zebrafish (*Danio rerio*).^2^^,^^3^ Researchers may apply this protocol to other small fish species and types of visual discrimination, provided that tank dimensions and stimulus presentation parameters are adjusted to match species-specific behavioral ecology and visual range.

Before performing the habituation/dishabituation paradigm:

1. Confirm ethical approval for all planned procedures with your institution’s animal care committee.

***Note:*** Approval should explicitly cover housing conditions, isolation duration, and the behavioral testing setup.

2. Prepare the stimulus presentation system (e.g., printed stimuli cards). Verify that stimulus size, contrast, and visibility are appropriate for the chosen species.

***Note:*** When adapting to zebrafish or another species, adjust stimulus visual angle to match the typical viewing distance of that species, considering the experimental corridor length (here, 20 cm). To calculate the visual angle under which the elements are seen one can use the formula $\alpha =\arctan\left(\frac{l/2}{d}\right)$ where *α* is the visual angle in radiants, arctan the invers tangent, *l* the image element dimension and d the viewing distance. For a conservative computation one can use as *l* the dimension of the smaller element and as *d* the maximum distance possible (here 20 cm). The visual angle should then be compared to, and exceed, the discrimination threshold of the species, as determined by appropriate studies and literature characterizing its visual system.

3. Prepare the behavioral recording system (e.g., monitor, tracking camera).

**CRITICAL:** Ensure recording settings allow continuous, good quality tracking of individual fish (adequate frame rate, no glare, stable focus; the important point is to manage fish position extraction at each time point).

4. Assemble and position the experimental corridors within the testing tank.

***Note:*** Dimensions may be scaled to match species size. Multiple corridors can be installed in parallel to increase throughput.

5. Prepare the behavioral testing room to maintain low ambient noise and stable illumination and temperature.

### Innovation

This protocol translates a classical and foundational paradigm in developmental and cognitive neuroscience, habituation/dishabituation, extensively used to probe perceptual discrimination in non-verbal human infants,^4^^,^^5^ into a fully aquatic model system. In traditional infant research, prolonged exposure to a constant stimulus leads to a measurable decline in attention (typically quantified as reduced looking time). A subsequent change of a relevant feature is then introduced during a single dishabituation test. If the subject detects the change, attention rebounds, revealing discrimination abilities without requiring any trained report.

We implement this framework in fish by replacing fixation-based measures with a direct, ethologically grounded metric: time spent in proximity to the stimulus across habituation and dishabituation phases. This modification preserves the logic and interpretability of the original paradigm while making it compatible with freely swimming animals.

By bringing a cornerstone human-infant methodology into fish research, the protocol provides a novel, scalable, and repeatable assay that directly assesses spontaneous discrimination in aquatic vertebrates. It not only expands the behavioral toolkit available for fish but also enables rigorous cross-species comparisons of perceptual and cognitive processing, linking mechanisms across vertebrate evolution with unprecedented experimental continuity.

### Institutional permissions

All experiments involving fish or other live vertebrates must be conducted in accordance with institutional and national ethical regulations. Although the behavioral procedures described here are non-invasive, temporary social isolation may cause mild distress. In our implementation, this is reduced by maintaining continuous exposure to conspecific olfactory cues; however, ethical approval is required. Our reference work^1^ for this protocol complied with all applicable national and European laws concerning the use of animals in research and was examined and approved by the Ethical Committee of the University of Trento (Organo Preposto al Benessere Animale, OPBA; Protocol n. 06/2024).

### Stimuli preparation

**Timing: Variable**

6. Design the visual stimuli.
  ***Note:*** In our case (numerical experiments), stimuli consist of square arenas containing arrays of different number of dots, with controlled spatial and geometric configurations.
  ***Note:*** Stimuli (square arenas) should measure 6 × 6 cm (this is the whole area available for stimuli, fitting the short side of the experimental corridor).
  a. Adjust dimensions if the corridor size changes.
    **CRITICAL:** Ensure that the entire stimulus remains fully submerged during testing (water depth = 7 cm in this protocol). See an example of experimental corridors with stimuli inserted in Figure 1A and an actual example of stimuli insertion in the movie at https://www.youtube.com/watch?v=k3STUNTB0Cw.

**Figure 1:**
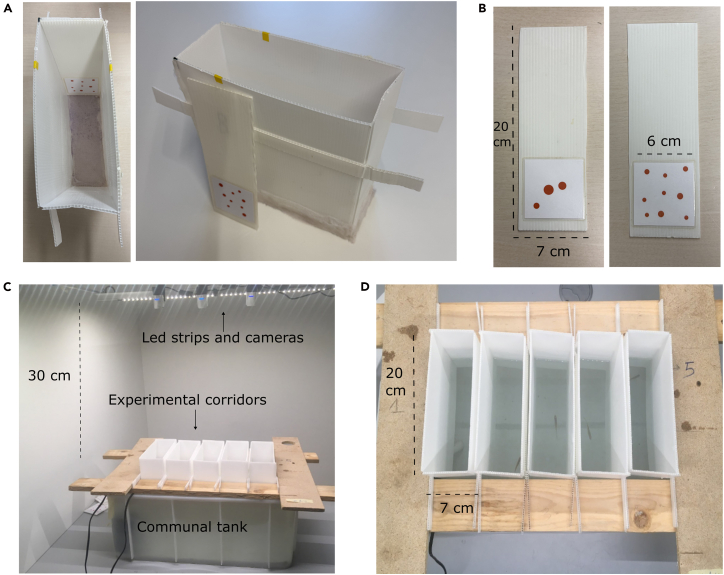
Setup (A) Experimental corridor with stimulus inserted on its shortest side. (B) Stimulus strip with laminated stimulus glued on the bottom extremity. (C) Accommodation tank with multiple corridors and recording system. (D) Example of fish view as recorded from the cameras on top of the experimental corridors.
  b. Create visual stimuli appropriately controlled depending on the experimental design.
    ***Note:*** Control conditions can be applied within subjects during habituation trials and between subjects during the dishabituation (test) phase.
    ***Note:*** In the present application to numerical discrimination in *Danionella*, stimuli consist of dot arrays differing in numerosity. Stimuli were generated in MATLAB using the GeNEsIS software^6^ and saved as.png files.
    ***Note:*** Stimuli should be controlled depending on the specific experiment. Our numerical stimuli are controlled for geometrical and spatial properties (total dot area, perimeter, dot radius, inter-dot distance, and convex hull). These features are continuously varied across habituation trials and sessions within each fish (within-subject design).
    **CRITICAL:** The feature of interest (here, numerosity) should remain constant during habituation. The dishabituation test phase consists of a single trial per fish, in which a novel stimulus (here, a new numerosity) is presented. Different control conditions for the dishabituation stimulus can be compared across different subjects (between-subject design), since multiple dishabituation trials per fish would disrupt the habituation effect.
7. Print and laminate stimuli. Figure 1B.
  a. Print stimuli on 160 g/m^2^ paper.
    ***Note:*** Use an appropriate weight to ensure flatness during lamination.
  b. Laminate the printed sheets and cut them precisely, ensuring that the laminate edges are fully sealed and free of gaps (to prevent water ingress and damage).
  c. Using a water-resistant adhesive, attach each laminated 6 × 6 cm stimulus to the lower portion of a 7 × 20 × 0.4 cm polypropylene strip.
  d. Verify that the final mounted stimulus fits snugly into the corridor, covering its short side without insertion problems.

### Experimental setup

**Timing: Variable**

8. Create experimental corridors. Figure 1A.
  a. Construct experimental corridors by cutting polypropylene sheets to the required dimensions.
    ***Note:*** In this protocol, the four sides of a corridor measures 20 × 7 × 15 cm: two long panels of 20 × 15 cm and two short panels of 7 × 15 cm.
  b. Carefully join all sides using water-resistant silicone.
    ***Note:*** Alternatively, adjacent sides can be formed from a single polypropylene sheet by folding it to create the appropriate angles.
  c. To close the bottom of the corridor, glue a fine-mesh net to the lower edges of the walls.
    ***Note:*** Corridor dimensions can be adapted for different species.
    **CRITICAL:** Ensure that the mesh size is small enough to prevent fish from escaping. Ensure that all joints and edges are thoroughly sealed with silicone to prevent holes or gaps where fish could hide or escape.
  d. On the upper part of the corridor, attach two horizontal supporting ledges extending outward from both sides.
    ***Note:*** This allows the corridor to hang stably by resting the ledges on the edges of the communal tank.
    **CRITICAL:** The corridor height should be designed so that, when suspended in the tank, the water level reaches the appropriate depth to fully submerge the stimuli (7 cm in this protocol).
9. Prepare experimental communal tank to accommodate multiple corridors. Figures 1C and 1D.
  ***Note:*** In this setup, corridors are suspended by their protruding supports resting on the edges of the communal tank.
  ***Note:*** The communal tank can accommodate multiple corridors (e.g., 5 in Figure 1C, within a 40L communal tank with recirculating, filtered water, supplemented with 10 mL “Sera aquatan” and 16 mL “Sera bio nitrivec”).
  ***Note:*** The whole setup is maintained at an average temperature of 28°C under a 14 h light / 10 h dark cycle.
  **CRITICAL:** Once the corridors are immersed, they should get filled with water to a height of 7 cm.
  a. Illuminate the tank using two lateral LED strips suspended approximately 30 cm above the water level, one along each long side.
    ***Note:*** This lateral dual-strip configuration prevents surface reflections caused by a single central overhead light source, which would otherwise interfere with video recording and reduce fish visibility.
  b. For video recording, position multiple cameras approximately 30 cm above the tank to capture the entire setup and connect them to the recording computer.
    ***Note:*** In our case, one camera is used to record two corridors.
    **CRITICAL:** Ensure that recording settings allow continuous, high-quality tracking of individual fish (sufficient frame rate, minimal glare, stable focus).
    ***Note:*** The size of the communal tank and the number of cameras can be adjusted depending on how many corridors (and therefore fish) are run in parallel.
    ***Note:*** Corridors can be stabilized blocking them from the sides with boards (see Figures 1C and 1D).

## Key resources table

**Table:** 

REAGENT or RESOURCE	SOURCE	IDENTIFIER
**Deposited data**		

Data for reference paper	Zanon et al.^1^	https://doi.org/10.6084/m9.figshare.28271237
R script for statistical data analysis of reference paper	Zanon et al.^1^	https://doi.org/10.6084/m9.figshare.28271237

**Experimental models: Organisms/strains**		

WT *Danionella Cerebrum*	Benjamin Judkewitz’s laboratory, Charité and Humboldt University, Berlin, DE	N/A

**Software and algorithms**		

RStudio version 2023.06.0+421	Posit Software, PBC	https://posit.co/download/rstudio-desktop/
Matlab version R2023b	MathWorks	https://it.mathworks.com/products/matlab.html
GeNEsIS (stimuli creation tool)	Zanon et al.^6^	https://github.com/MirkoZanon/GeNEsIS
DeepLabCut (tracking tool)	Mathis et al.^7^	https://deeplabcut.github.io/DeepLabCut/README.html

**Other**		

Microsoft LifeCam Studio camera	Microsoft - Farnell distributor	Code Q2F-00015
LED 1459 lm/m 4000K, 13W/m Strip 2835 Honglitronic	Orizzonte Led Lighting	Code OR40sF52060821
Flexible white corrugated polypropylene sheet (3.5 mm thickness)	Polionda® (Ondaplast S.p.A.)	Code 007067
Fine-mesh net (100 μm)	MAXSPECT	Cat. PCT-MB4B, EAN 6971764810621
Aquarium Amtra Wave Zen Artist 40L tank	Amtra	Code A2001067
Water-resistant aquarium-safe silicone sealant (AquaSil Transparent)	JBL	Cat# 6139100
Water conditioner (Sera Aquatan)	Sera GmbH	Cat# 03030-03080
Biological filter starter (Bio nitrivec)	Sera GmbH	Cat# 03730
Aquarium heater (50W)	JBL	Art.n. 6042200
Internal Filter Sera fil 60	Sera GmbH	Cat# 06843

## Step-by-step method details

### Familiarization phase

**Timing: 2 days**

This section establishes a stable and minimally stressful testing environment by allowing fish to acclimate to the experimental corridors before behavioral testing. The familiarization period is designed to reduce novelty- and handling-related stress, ensure consistent environmental exposure across subjects, and stabilize baseline behavior prior to the habituation/dishabituation protocol.

1. Place each fish in an individual corridor two days before the start of the experiment to allow familiarization with the testing environment.
  a. Suspend all corridors in the communal tank and position one fish per corridor, so that fish remain physically isolated but share the same circulating water and chemical cues from conspecifics.
    ***Note:*** This reduces isolation-related distress while avoiding the disturbance that would result from repeatedly transferring fish.
  b. Ensure that corridors are fully submerged at the proper water depth (here, 7 cm) and that no experimental stimuli are present during this period.
2. Maintain each fish in the same corridor for the entire duration of the familiarization and experiment.
  a. From now on, avoid moving fish out from corridors, as relocation increases stress and disrupts habituation if actuated between experimental sessions.
  b. Monitor fish daily to confirm normal activity and absence of distress.
  c. Maintain standard tank conditions, using the water circulation and keeping constant water temperature and light/dark cycles.
  d. Feed fish normally in their corridors and remove any excess food to maintain water quality.
3. Let the fish familiarize with this setup for 2 days before starting the habituation phase. Troubleshooting 1.

### Habituation sessions

**Timing: 5 days (2 h/day)**

This section describes the repeated stimulus-exposure phase aimed at inducing habituation to a constant target feature (here, numerosity) while varying non-informative visual parameters. By repeatedly presenting stimuli that share the same numerosity but differ in control features, this phase reduces responsiveness to low-level perceptual factors and establishes a stable habituated baseline, enabling reliable detection of stimulus-specific recovery of response during the subsequent dishabituation test.

4. Prepare all stimuli in advance for the entire habituation phase. Troubleshooting 2.
  a. Keep the variable of interest (here, numerosity) constant during the whole habituation and vary control features (here, dot size, spacing, convex hull) across trials and days.
    ***Note:*** This variation during habituation trials allows subjects to lose responsiveness to factors of no interest (e.g., luminance, which can be controlled via total element area, spacing, and convex hull), while preserving responsiveness to numerosity (kept constant during habituation). If change in numerosity is perceived, a response will re-emerge in the final dishabituation trial when it is modified.
  b. Arrange stimuli in labeled, day-by-day and trial-by-trial order for each fish/corridor to allow seamless progression.
5. Conduct the daily habituation session (six trials per day, ∼2 h total). Troubleshooting 1.
  a. Start the recording.
  b. Insert the correct stimulus into the short corridor side (the same side is maintained through all the experiment sessions).
  c. Allow the trial to run for 20 min (just recording the fish freely moving).
    **CRITICAL:** Do not disturb the fish during this time; avoid sudden noises, vibrations, or movements of the tank or surrounding equipment. Avoid ambient light modifications.
  d. At the end of the 20-minute period, stop the recording and save the video file with an informative filename (e.g., *FishID_DayX_TrialX*).
  e. Immediately proceed to the next trial by repeating the previous steps (5a–d).
    **CRITICAL:** Do not feed the fish between trials. Keep trials consistently subsequent without pauses.
  f. Run a total of 6 habituation trials per day.
6. Repeat the daily habituation procedure (step 5) for four consecutive days (Days 1–4).

***Note:*** Keep the time of day consistent across all days to avoid circadian variability (e.g., run all sessions between 10:00 and 12:00).

**CRITICAL:** Use six different configurations of the same feature (here, numerosity) each day; vary control configurations across days according to the planned stimuli schedule.

**CRITICAL:** Maintain each fish in the same corridor throughout.

7. End-of-day routine. Troubleshooting 3.
  a. After completing the sixth trial, confirm that all video files are saved and correctly labeled.
  b. Feed the fish in their corridors.
  c. Check fish for normal behavior before concluding the session.

### Dishabituation session

**Timing: 2 h**

This section defines the critical test phase used to assess stimulus feature discrimination. By maintaining procedures identical to the habituation sessions and altering only the target variable (here, numerosity) in the final trial, this phase evaluates whether a change in the variable of interest elicits a recovery of behavioral response, thereby confirming that habituation was feature-specific rather than due to general fatigue or nonspecific adaptation.

***Note:*** This last daily session (Day 5) is completely identical to all the previous ones (habituation sessions, Days 1–4) from a procedural point of view, the only difference lays in the type of stimulus used in the very last trial (therefore defined as ‘dishabituation trial’).

8. Prepare all stimuli in advance for the entire habituation/dishabituation phase. Troubleshooting 2.
  a. Keep the variable of interest (here, numerosity) constant during the habituation trials, while it should be changed in the last dishabituation trial and vary control features (here, dot size, spacing, convex hull) across trials.
  b. Arrange stimuli in labeled, trial-by-trial order for each fish/corridor to allow seamless progression.
9. Conduct the daily session for Day 5 (five habituation trials + one dishabituation trial, ∼2 h total). Troubleshooting 1.
  a. Start the recording.
  b. Insert the correct stimulus into the short corridor side.
  c. Allow the trial to run for 20 min (just recording the fish freely moving).
    **CRITICAL:** Do not disturb the fish during this time; avoid sudden noises, vibrations, or movements of the tank or surrounding equipment. Avoid ambient light modifications.
  d. At the end of the 20-minute period, stop the recording and save the video file with an informative filename (e.g., *FishID_DayX_TrialX*).
  e. Immediately proceed to the next trial by repeating the previous steps (9a-d).
    **CRITICAL:** Do not feed the fish between trials. Keep trials consistently subsequent without pauses.
  f. Run a total of 5 habituation trials followed by a final dishabituation test trial, maintaining each fish in the same corridor throughout.
    ***Note:*** Keep the time of day consistent with previous habituation sessions to avoid circadian variability (e.g., run it between 10:00 and 12:00).
    **CRITICAL:** Use five different configurations of the same feature (here, numerosity) for the habituation; present a new experimental feature (here, new numerosity) in the final dishabituation trial.
10. End-of-day routine. Troubleshooting 3.
  a. After completing the sixth trial, confirm that all video files are saved and correctly labeled.
  b. Feed the fish and free them in their home tanks. Experiment is concluded.

## Expected outcomes

Following this protocol, researchers can expect to obtain video recordings of individual fish freely exploring the experimental corridors across the habituation phase and the final dishabituation trial. From these recordings, the time spent by each fish in proximity to the stimulus is extracted, yielding a relative percentage of time spent near the stimulus for each trial.

**Figure 2:**
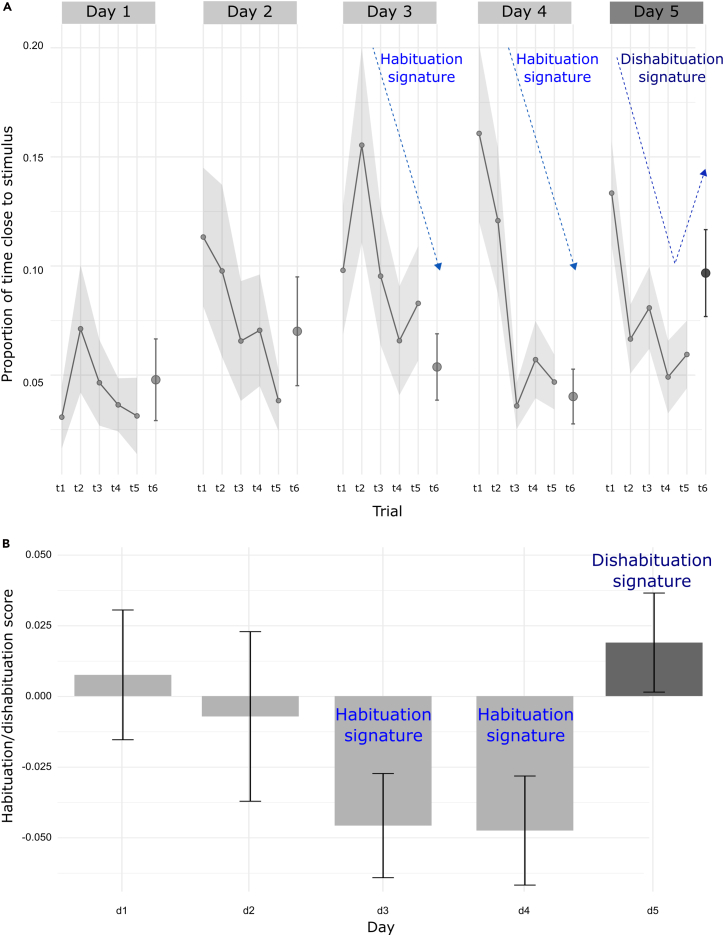
Experimental output: habituation/dishabituation example curves from Zanon et al.^1^ (A) Habituation/dishabituation curves with proportion of time spent by fish in the area close to the stimulus (3 cm) reported in the y axis. Data are shown across the 6 daily trials (x axis) and the 5 experimental days. Only in the last (6^th^) trial of last day (5^th^), the stimulus feature of interest is varied to test for dishabituation, which can be inferred by an increase in the time spent by fish close to the stimulus. (Lines with shadowed error bars represent mean with SEM across subjects for trials 1 to 5; for final trial6 SEM is separately represented as a single error bar). (B) Differential scores (y axis) across the 5 experimental days (x axis). A negative score indicate habituation, while a positive one is proxy for dishabituation. In this example it is clear how fish get fully habituated already after 2 days of exposure to a specific numerosity, while at day 5 they show dishabituation to the new numerosity presented in the last trial. Bars and error bars represent subjects mean and SEM.

During the habituation phase, fish are expected to progressively decrease their investigation of the repeated stimulus, reflected as a reduction in approach or attention behaviors across consecutive trials. Although some novelty-driven response may occur at the beginning of each day, the overall habituation trend should become increasingly evident from the second to the sixth trial within a day and across days. Figure 2A.

In the dishabituation trial, the presentation of a novel stimulus (here, a new numerosity) should elicit a measurable increase in investigation (e.g., increased time spent near the stimulus), indicating that the fish discriminates between the familiar and the novel feature. Figure 2B. See an example in the movie at https://www.youtube.com/watch?v=k3STUNTB0Cw.

The protocol is designed to minimize stress and other confounding factors, enabling consistent and reproducible behavioral outcomes across individuals. Maintaining stable environmental conditions and standardized recording settings ensures that the resulting video data are suitable for fish tracking and quantitative analyses, producing reliable datasets for statistical comparison.

Overall, this approach yields behavioral data capturing both habituation and dishabituation responses and provides a robust framework for assessing perceptual discrimination in small fish species such as *Danionella* or zebrafish.

## Quantification and statistical analysis

Video recordings are analyzed to quantify fish position relative to the stimulus. Position can be scored manually by timing how long the fish remains in proximity to the stimulus or automatically using a tracking tool such as DeepLabCut to label and track the fish head. The fish is considered “close to the stimulus” when located within the 15% of the corridor length adjacent to the stimulus-side wall (here, with a 20 cm corridor, the stimulus zone corresponds to the 3 cm nearest to the stimulus; the choice area was defined as approximately two average body lengths for *Danionella*).

Because habituation can also occur within the single 20-minute trial, only the first 180 seconds of each trial are analyzed. The primary measure is the proportion of time spent in the stimulus zone:

Proportionoftimenearstimulust=(Secondsspentinstimuluszone)/180

To assess habituation and dishabituation at the day level, a differential score is calculated by subtracting the average proportion of time near the stimulus across Trials 1–5 from the value in Trial 6, for each day:

Differentialscore=(tTrial6)–(MeantofTrials1–5)

This score ranges from −1 to +1. Strongly negative values indicate reduced investigation in Trial 6 (habituation); values near zero indicate stable behavior; strongly positive values indicate increased investigation in Trial 6 (dishabituation).

This calculation is applied across all five days. Differential scores from Days 1–4 are interpreted as *habituation scores*, while the Day 5 score (where the stimulus is changed) can be referred to as *dishabituation score*.

Habituation is evidenced by progressively decreasing scores across Days 1–4, trending negative by Day 4. Dishabituation is indicated by a positive score on Day 5, and by a significant difference between Day 4 and Day 5 scores.

More specifically, to test habituation, the Day 4 differential score is compared against zero using a Wilcoxon signed-rank test or one-sample *t* test (a significantly negative value indicates habituation). To test dishabituation, differential scores from Day 4 and Day 5 are compared statistically, for example using a permutation ANOVA (e.g., *aovperm*, *permuco* R package, 5000 permutations), with Day (4 vs. 5) and Control Type as factors. Control Type distinguishes possible stimulus presentation controls (e.g., here, habituation to 3 and dishabituation with 9, or vice-versa, enabling assessment of directional effects in numerosity).

Fish are excluded if they remain immobile for more than 50% of the analyzed time, show signs of distress, or if technical issues prevent clear and reliable tracking (usually rare, less than 5% rate).

Note that, from a statistical standpoint, the limitation of a single dishabituation trial per session is addressed by using a between-subjects design. This is essential for maintaining a clear and uncontaminated dishabituation effect.

## Limitations

This protocol relies on maintaining consistent environmental and experimental conditions, and deviations can affect the reliability of results. Fish behavior may be influenced by stress, handling, or environmental fluctuations such as temperature, lighting, or water quality. Although the communal water immersion reduces isolation stress, individual differences in response to novel stimuli may still introduce variability. The protocol assumes that all fish can habituate to repeated stimulus exposure; species or developmental stages with very high sensitivity or low activity levels may not show clear habituation or dishabituation effects. The physical setup also imposes constraints: corridor dimensions, camera positioning, and barrier integrity must be precise to ensure consistent stimulus exposure and prevent escapes. Corridor dimensions should be adapted to the fish size to allow free movement, exploration, and the option to stay away from the stimulus. Additionally, the protocol is designed for single dishabituation trials to detect novelty responses; repeating dishabituation trials within subjects may reduce novelty effects and weaken interpretability. Finally, tracking accuracy depends on video quality, lighting, and camera resolution, which can limit automated analysis if not properly optimized.

## Troubleshooting

### Problem 1

Fish stress, abnormal behavior, or escape/hiding (Steps 3, 5, 9):

Fish display freezing, frantic swimming, hiding, or attempt to escape corridors, potentially disrupting habituation.

### Potential solution

Maintain fish immersed in communal tank water to reduce isolation stress. Confirm all silicone seals and net/grid barriers are intact. Avoid disturbing fish during trials. Keep handling and environmental conditions consistent. If fish show difficulty in habituate to the stimuli, the number of habituation days could also be increased. If instead fish show consistent distress behaviour, remove the fish from the setup and discard the recordings.

### Problem 2

Stimulus preparation and insertion (Steps 4, 8):

Stimuli are damaged by water or incorrectly sealed, leading to inconsistent presentation.

### Potential solution

Ensure laminated cards are fully sealed with no holes and that water-resistant glue properly attaches them to the polypropylene strip. Inspect stimuli before each session and keep spare pre-laminated cards ready.

### Problem 3

Recording quality issues (Steps 7, 10):

Videos have glare, poor focus, or low frame rate, making tracking unreliable.

### Potential solution

Adjust camera angle, distance, and lighting before starting the experiment. Test the recording settings during a pilot session and keep all camera positions fixed throughout the full protocol.

***Note:*** Extremely high video resolution is not strictly required; the key is that the fish remains clearly visible and segmentable at all times. Higher resolution can be used if more detailed analyses (e.g., head tracking, visual angle measurement) are needed. Overall, as a rule of thumb, as long as the fish is visually detectable in each frame, the resolution is sufficient for both manual and automatic scoring.

### Problem 4

Sources of variability in habituation/dishabituation:

Variations in feeding, session timing, or daily routines can affect fish behavior and habituation outcomes.

### Potential solution

Feed fish only after completing all trials each day. Keep the time of day consistent for all sessions (e.g., 10:00–12:00) and maintain stable environmental conditions.

### Problem 5

Protocol should be adapted to different species, dimensions, and other constraints.

### Potential solution

Alternatives and flexibility:

• Corridor and stimuli dimensions can be adjusted for different species, provided water immersion and proper image features discrimination are maintained.
• Camera type, number, and positioning can vary as long as the entire setup is captured at sufficient resolution and frame rate for accurate tracking.
• Laminating materials or adhesives can be replaced with functionally equivalent, water-resistant options, ensuring stimuli remain intact.

## Resource availability

### Lead contact

Further information and requests for resources should be directed to and will be fulfilled by the lead contact, Mirko Zanon (mirko.zanon@unitn.it).

### Technical contact

For technical specifics on executing the protocol, Dr. Mirko Zanon (mirko.zanon@unitn.it) will provide support to ensure its correct implementation.

### Materials availability

This study did not generate new unique reagents and materials.

### Data and code availability

• Data related to the original reference work^1^ are available in Figshare repository: https://doi.org/10.6084/m9.figshare.28271237.
• Code used for the analysis of the original reference work^1^ is available in Figshare repository: https://doi.org/10.6084/m9.figshare.28271237.

## Acknowledgments

This project was funded by ERC European Union’s Horizon 2020 research and innovation program grant agreement 833504 – SPANUMBRA to G.V.; by FARE–Ricerca in Italia: Framework per l’Attrazione ed il Rafforzamento delle Eccellenze per la ricerca in Italia, III edizione, project “NUMBRISH–The neurobiology of numerical cognition: searching for a molecular genetic signature in the zebrafish brain” Prot. R20YL9WN9N to G.V.; and by PRIN – Progetti di rilevante Interesse Nazionale 2022 – PNRR grant agreement P2022TKY7B: “The emergence of proto-arithmetic abilities with empty and non-empty sets” to G.V.

## Author contributions

Original conceptualization, G.V.; method implementation, M.Z., D.P., and A.M.; protocol writing, M.Z.; supervision, G.V.; funding, G.V. All authors contributed to the final version of this work.

## Declaration of interests

The authors declare no competing interests.

## References

[1] Zanon M., Fraser S.E., Vallortigara G. (2025). Numerical discrimination in Danionella. iScience.

[2] Messina A., Potrich D., Schiona I., Sovrano V.A., Fraser S.E., Brennan C.H., Vallortigara G. (2020). Response to change in the number of visual stimuli in zebrafish:A behavioural and molecular study. Sci. Rep..

[3] Messina A., Potrich D., Schiona I., Sovrano V.A., Fraser S.E., Brennan C.H., Vallortigara G. (2022). Neurons in the Dorso-Central Division of Zebrafish Pallium Respond to Change in Visual Numerosity. Cereb. Cortex.

[4] Colombo J., Mitchell D.W. (2009). Infant visual habituation. Neurobiol. Learn. Mem..

[5] Clifton R.K., Nelson M.N. (2016). Habituation: Perspectives from Child Development, Animal Behavior, and Neurophysiology.

[6] Zanon M., Potrich D., Bortot M., Vallortigara G. (2022). Towards a standardization of non-symbolic numerical experiments: GeNEsIS, a flexible and user-friendly tool to generate controlled stimuli. Behav. Res..

[7] Mathis A., Mamidanna P., Cury K.M., Abe T., Murthy V.N., Mathis M.W., Bethge M. (2018). DeepLabCut: markerless pose estimation of user-defined body parts with deep learning. Nat. Neurosci..

